# PD-L1 expression and presence of TILs in small intestinal neuroendocrine tumours

**DOI:** 10.18632/oncotarget.24464

**Published:** 2018-02-12

**Authors:** Angela Lamarca, Daisuke Nonaka, Wolfgang Breitwieser, Garry Ashton, Jorge Barriuso, Mairéad G. McNamara, Sharzad Moghadam, Jane Rogan, Wasat Mansoor, Richard A. Hubner, Christopher Clark, Bipasha Chakrabarty, Juan W. Valle

**Affiliations:** ^1^ Department of Medical Oncology, The Christie NHS Foundation Trust, Manchester, UK; ^2^ Department of Histopathology, The Christie NHS Foundation Trust, Manchester, UK; ^3^ Division of Molecular and Clinical Cancer Sciences, Faculty of Biology, Medicine and Health, University of Manchester, Manchester, UK; ^4^ Molecular Biology Core Facility, Cancer Research UK Manchester Institute, Manchester, UK; ^5^ Manchester Cancer Research Centre (MCRC) BioBank, University of Manchester, Manchester, UK

**Keywords:** PD-L1, tumour infiltrating lymphocytes, neuroendocrine, immunotherapy, treatment

## Abstract

**Background:**

The extent of resistance to immune surveillance in patients with well-differentiated (Wd) (grade 1/2) small-intestinal neuroendocrine tumours (Si-NETs) is unknown.

**Methods:**

Patients diagnosed with Wd Si-NETs (excluding appendix, which are considered to have a different biology to other midgut NETs) were eligible. Tumoural programmed death (PD)-ligand(L) 1 (PD-L1)/PD-L2/PD-1 and tumour infiltrating lymphocytes (TILs) [presence and phenotype] were analysed in archival tissue by immunohistochemistry (IHC); reverse transcription quantitative polymerase chain reaction (RT-qPCR) was used for confirmation of IHC results.

**Results:**

Of 109 patients screened, 62 were eligible: 54.8% were male; median age was 63.7 years (95%-CI 59.7-67.2); disease stage II: 4.8%, III: 40.3% and IV: 54.8%; 41.9% were functional. Analysed samples (67.1% from primary tumours, 32.9% from metastases) were of grade 1 (67.1%) or 2 (32.86%) with a median Ki-67 of 2%. From the total of 62 eligible patients, 70 and 63 samples were suitable for IHC and RT-qPCR analysis, respectively. PD-L1 expression within tumour cells and TILs were identified in 12.8% and 24.3% of samples respectively; 30% of samples showed PD-L1 expression within tumour cells and/or TILs. PD-1 was present in TILs in 22.8% of samples. Majority of samples showed significant presence of CD4^+^ (focal 42.86%; moderate 2.86%) and CD8^+^ (focal 92.86%; moderate 4.29%) TILs. IHC findings were confirmed with RT-qPCR; which showed higher expression levels of PD-L1 (p-value 0.007) and PD-1 (p-value 0.001) in samples positive for IHC compared to negative-IHC.

**Conclusions:**

Thirty-percent of patients express PD-L1 within tumour cells and/or TILs. Identification of presence of TILs was also significant and warrant the investigation of immunotherapy in this setting.

## INTRODUCTION

Gastro-entero-pancreatic (GEP) neuroendocrine neoplasms (NENs) are relatively rare, although the incidence has been rising during recent years [1]. The grade of the neoplasms is determined by tumour morphology along with the proliferation (Ki-67) index (assessed with the MIB1 antibody) and the mitotic index according to World Health Organisation (WHO) classification [2, 3]. Overall, 20% of patients with well-differentiated (Wd) neuroendocrine tumours (NETs) present with distant metastases at initial diagnosis, with an estimated median overall survival between 2 and 5 years depending on the series [1]. For patients with advanced Wd small intestine NETs (Si-NETs), first-line treatment with a somatostatin analogue (SSA; either lanreotide [4] or octreotide [5]) is currently considered the standard of care. Although options of treatment on progression on SSAs are emerging, such as Peptide Receptor Radionuclide Therapy (PRRT)[6], everolimus (for non-functional NET patients)[7] or chemotherapy (for selected patients)[8]; new options for therapy are required.

Despite the large number of tumour antigens induced by genetic and epigenetic changes found in all cancers, tumours are able to develop resistance to immune surveillance by inducing tolerance among tumour-specific T-cells and by expressing ligands that engage inhibitory receptors and dampen T-cell functions within the tumour microenvironment. This mechanism has been previously identified as one of the hallmarks of cancer [9]. Targeting inhibition of the immune system, mainly in the form of anti-cytotoxic T-lymphocyte-associated antigen 4 (CTLA4) or immune check-point inhibitors, has been successfully established as a treatment for patients with solid tumours such as melanoma [10–14], non-small cell lung cancer [15–17], squamous cell head and neck [18, 19] cancer and Merkel cell carcinoma [20] with an acceptable safety profile [21, 22]. This new-generation of immunotherapy treatments (i.e. CTLA4 or immune check-point inhibition) has not yet been proven to be effective in patients with NETs [23, 24]; clinical trials focused on NETs are still recruiting patients (www.clinicaltrials.gov; last accessed 26^th^ July 2017: NCT03167853, NCT02955069, NCT03095274). In contrast, interferon (IFN), a classic immunotherapy drug, has the ability to stimulate T-cell function, control the secretion of tumour products and inhibit tumour growth [25] by activation of the T-cell response against the tumour and angiogenesis inhibition [26, 27] in NETs. IFN has been shown to reduce symptoms related to hormone secretion in 40-50% of patients with NETs, resulting in a radiological partial response rate of 10% with disease stabilisation seen in 20-40% [28].

CTLA-4, an inhibitory receptor that down-modulates the initial stages of T-cell activation, was the first clinically-validated check-point pathway target [29]. Programmed death-1 (PD-1) protein is another T-cell co-inhibitory receptor with a structure similar to that of CTLA-4, but with a distinct biologic function and ligand specificity [30]. PD-1 has two known ligands; PD-L1 (B7-H1) and PD-L2 (B7-DC). Blockade of the interaction between PD-1 and PD-L1 potentiates immune responses *in vitro* and mediates preclinical anti-tumour activity [31–33].

PD-L1 is the primary PD-1 ligand that is up-regulated in solid tumours, where it can inhibit cytokine production and the cytolytic activity of PD-1^+^, tumour-infiltrating CD4^+^ and CD8^+^ T-cells. These properties make PD-L1 a potentially promising target for cancer immunotherapy. However, the role of PD-L1 expression in the tumour is far from being widely validated as a general predictive biomarker [13]. The main key for understanding inter-tumoural differences in response appears to be the expression of the PD-1 ligands: PD-L1 and PD-L2 in the tumour microenvironment [34]. Preliminary evidence suggests that the expression of PD-L1 may indeed select for patients with an improved response to PD-1 axis inhibitors. Expression of PD-L1/2 has been analysed in some NEN subgroups, including poorly-differentiated (grade 3) neuroendocrine carcinomas (NECs)[35, 36], with reported PD-L1 expression rates which vary between 20-50%, however there is very limited data describing PD-L1/2 expression in well-differentiated NETs, including Si-NETs [37, 38]

This study aimed to assess the check-point pathway protein expression in patients with well-differentiated Si-NETs, with a view to explore the potential role for immunotherapy.

## RESULTS

Out of a total of 109 patients screened, 62 patients had available samples for analysis (Supplementary Figure 1). Out of these 62 patients, 70 and 63 samples were available for IHC and RT-qPCR, respectively.

### Baseline characteristics

The median age at first diagnosis was 63.75 years (range 26.17-85.93) with a male/female ratio of 1:1. Most patients were of Eastern Cooperative Oncology Group performance status (ECOG-PS) 0 (43.55%) or 1 (48.39%). Carcinoid syndrome was present in 26 patients (41.94%): evident by flushing (24.19%), diarrhoea (30.65%) and/or wheezing (4.84%). Other baseline characteristics, such as past medical history of systemic inflammatory disease, are summarised in Table 1. Results of blood biomarkers are available in Supplementary Table 1.

**Table 1:** Patients’ baseline characteristics

Variable		Frequency (total n=62)	%
**Gender**	Female	28	45.16
	Male	34	54.84
**Age at first diagnosis (years)**	Median (range)	63.75	26.17-85.93
**Comorbidities (ACE-27)**	None	16	25.81
	Mild	41	66.13
	Moderate	5	8.06
**PMH of systemic inflammatory disease**	No	54	87.10
	Yes	8	12.90
**ECOG-PS**	0	27	43.55
	1	30	48.39
	2	4	6.45
	3	1	1.61
**Carcinoid syndrome**	Yes (Any symptom)	26	41.94
	Flushing	15	24.19
	Diarrhoea	19	30.65
	Wheezing	3	4.84
**TNM (ENETS)**	II	3	4.84
	III	25	40.32
	IV	34	54.84
**T (primary tumour)**	2	6	9.68
	3	20	32.26
	4	20	32.26
	X	16	25.81
**N (lymph node)**	0	2	3.23
	1	43	69.35
	X	17	27.42
**M (distant metastases)**	0	28	45.16
	1	34	54.84
**Number of sites of metastases**	1	19	30.65
	2	11	17.74
	3	4	6.45
**Site of metastases**	Distant mesenteric lymph nodes	16	25.81
	Liver	24	38.71
	Lung	1	1.61
	Peritoneum	9	14.52
	Bone	2	3.23
	Pancreas	1	1.61
	Ovary	1	1.61

ECOG-PS: Eastern Cooperative Oncology Group Performance Status score; 95%-CI: 95% confidence interval; NET: neuroendocrine tumour; PMH: past medical history; ACE-27: Adult Comorbidity Evaluation (ACE)-27 index; ENETS: European Neuroendocrine Tumour Society; X: not evaluated.

### Patient management

The stage at diagnosis was as follows: stage II (4.84%), stage III (40.32%) and stage IV (54.84%) (Table 1). Most patients had resection of the primary small-bowel tumour at some point during their management (52 patients; 83.87%); out of the 62 patients included, 51.61% had surgery performed with curative intent (Supplementary Table 2).

The median follow-up for the whole series of 62 patients was 55.22 months. By the end of the follow up period, 11 patients had died (17.74%); estimated median OS was 195.34 months (95%-CI 106.34-not reached). Estimated median RFS for patients treated with curative surgery (32 patients) and PFS for those treated with palliative intent (30 patients) were 62.49 months (95%-CI 25.42-142.49) and 49.33 months (95%-CI 29.23-64.83), respectively (Supplementary Table 2). Details of first-line therapy for patients treated with palliative intent and radiological response are summarised in Supplementary Table 1.

### Tumour characteristics

A total of 70 tissue samples were retrieved from the 62 patients eligible for this study: 47 were primary tumours and 23 were from metastatic sites (Table 2). Most (67.14%) tumours were grade 1 with a median Ki-67 of 2% (range 0.7-18%). The median maximum tumour diameter (mm) and surface (mm^2^) were 12 and 91, respectively. Out of the 62 patients, one patient had 4 samples available, 5 patients had 2 samples available, and the remaining 56 had only one tumour sample retrieved.

**Table 2:** Characteristics of retrieved samples

Variable		Frequency (total n=70 samples)	%
**Grade**	Grade 1	47	67.14
	Grade 2	23	32.86
**Ki67**	Median (range)	2 (0.7-18)	
**Maximum tumour diameter in analysed sample (mm)**	Median (range)	12 (2-30)	
**Maximum tumour surface (product diameters) in analysed sample (mm**^2^**)**	Median (range)	91 (3-600)	
**Medication before biopsy**	No	55	78.57
	Yes	15	21.43
	Chemotherapy	5^*^	7.14
	SSA	5	7.14
	Steroids	3	4.29
	IFN	2	2.86
**Sample type**	Primary tumour	47	67.14
	Metastatic site	23	32.86
	Liver	19	27.14
	Peritoneum	2	2.86
	Mesenteric mass	1	1.43
	Ovarian metastases	1	1.43

^*^ 4 of these 5 samples are from to the same patient.

IHC: immunohistochemistry; 95%-CI: 95% confidence interval; mm: millimetres; mm^2^: squared millimetres; SSA: somatostatin analogues; IFN: interferon.

Fifteen samples (21.43%) were taken while patients were on concomitant medications which could potentially affect the tumour immune infiltrate. These included SSAs (5 samples), chemotherapy (5 samples from 2 patients, as 4 samples were from one patient having multiple liver metastasectomies), steroids (3 samples) and IFN (2 samples).

### PD-1, PD-L1 and PD-L2 expression by IHC

**Figure 1:**
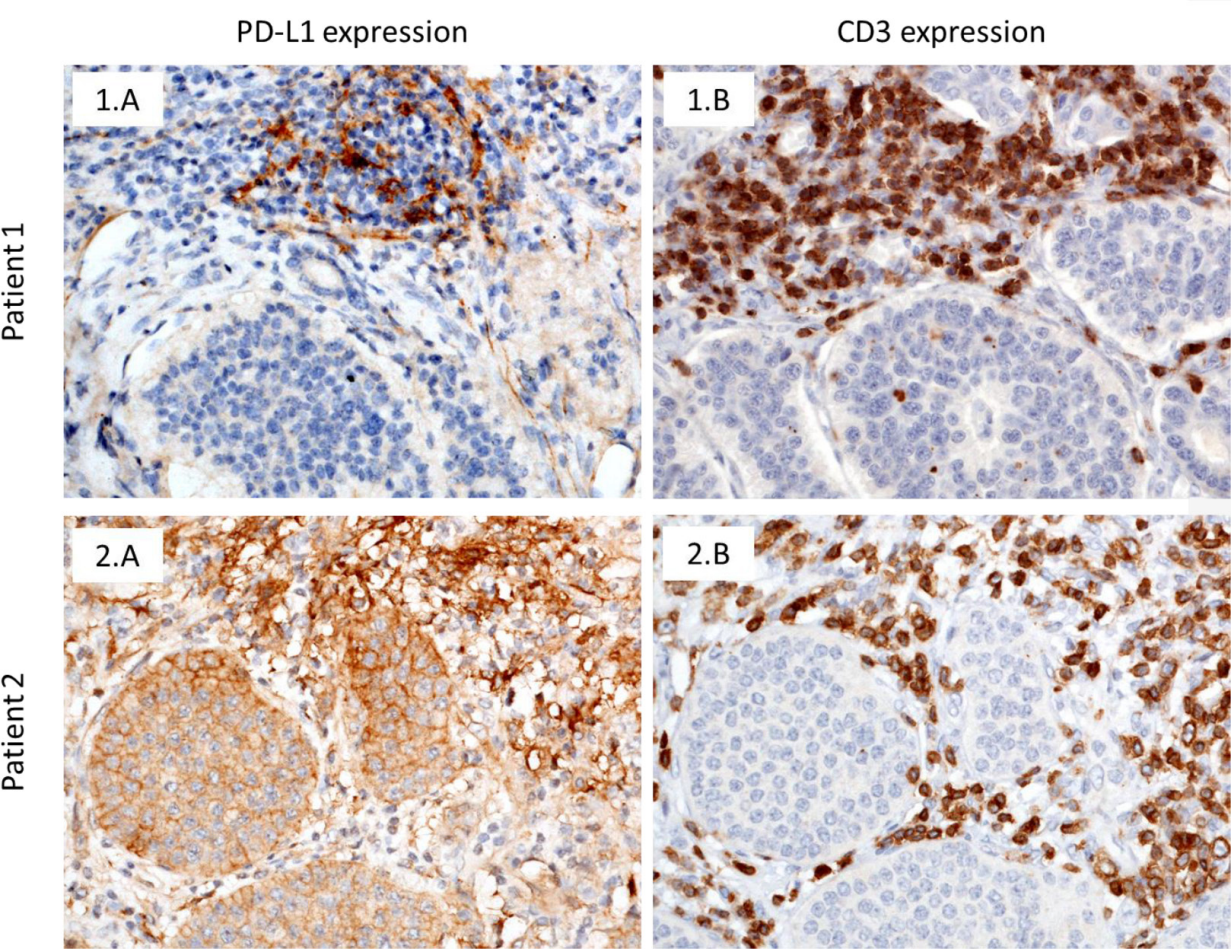
IHC assessment of FFPE archival tissue samples (x200 magnification) Patient 1: Tumour was negative for PD-L1 **(1.A)**; there were CD3 positive tumour infiltrating lymphocytes **(1.B)**. Patient 2: PD-L1 stain demonstrates membranous expression of tumour cells as well as infiltrating immune cells (the immune cells show strong expression while the expression in the tumour is variable in intensity **(2.A)**); CD3+ tumour infiltrating lymphocytes **(2.B)**. PD-L1: Programmed death-ligand 1; IHC: immunohistochemistry; FFPE: formalin-fixed paraffin embedded.

PD-L1 expression within tumour cells and TILs was identified in 12.8% and 24.3% of samples, respectively. However statistically significant (p-value 0.0083), correlation between PD-L1 expression within tumour cells and TILs was weak (rho Spearman correlation coefficient 0.313). PD-1 was present in TILs in 22.8% of samples. Even though the assay worked for the positive control tissue employed, no IHC expression of PD-L2 was identified in any samples. Lymphoid aggregates were identified in 19 samples (27.14%). The majority of samples showed significant presence of CD4^+^ (focal 42.86%; moderate 2.86%) and CD8^+^ (focal 92.86%; moderate 4.29%) TILs. See Figure 1 and Table 3 for further details.

**Table 3:** IHC expression of PD-1, PD-L1, PD-L2 and TILs

Cell type explored	Grade of expression	PD-L1 expression		PD-L2 expression		PD-1 expression		Lymphoid aggregates		CD3		CD68		CD4		CD8		CD20		CD4/CD8 ratio (applicable only for samples with CD4 and CD8 expression)	
		Freq	%	Freq	%	Freq	%	Freq	%	Freq	%	Freq	%	Freq	%	Freq	%	Freq	%	Freq	%
**Tumour cells**	No	61	87.14	70	100	n/a	n/a	n/a	n/a	n/a	n/a	n/a	n/a	n/a	n/a	n/a	n/a	n/a	n/a	n/a	n/a
	Yes	9	12.86	0	0	n/a	n/a	n/a	n/a	n/a	n/a	n/a	n/a	n/a	n/a	n/a	n/a	n/a	n/a	n/a	n/a
	0%	61	87.14	n/a	n/a	n/a	n/a	n/a	n/a	n/a	n/a	n/a	n/a	n/a	n/a	n/a	n/a	n/a	n/a	n/a	n/a
	5%	7	10.00	n/a	n/a	n/a	n/a	n/a	n/a	n/a	n/a	n/a	n/a	n/a	n/a	n/a	n/a	n/a	n/a	n/a	n/a
	10%	0	0	n/a	n/a	n/a	n/a	n/a	n/a	n/a	n/a	n/a	n/a	n/a	n/a	n/a	n/a	n/a	n/a	n/a	n/a
	20%	2	2.86	n/a	n/a	n/a	n/a	n/a	n/a	n/a	n/a	n/a	n/a	n/a	n/a	n/a	n/a	n/a	n/a	n/a	n/a
**TILs**	No	53	75.71	70	100	52	74.29	51	72.86	3	4.29	3	4.29	38	54.29	2	2.86	42	60.00	n/a	n/a
	Yes	17	24.29	0	0	18	25.71	19	27.14	67	95.71	67	95.71	32	45.71	68	97.14	28	40.00	n/a	n/a
	0%	53	75.71	n/a	n/a	n/a	n/a	n/a	n/a	n/a	n/a	n/a	n/a	n/a	n/a	n/a	n/a	n/a	n/a	n/a	n/a
	5%	15	21.43	n/a	n/a	n/a	n/a	n/a	n/a	n/a	n/a	n/a	n/a	n/a	n/a	n/a	n/a	n/a	n/a	n/a	n/a
	10%	1	1.43	n/a	n/a	n/a	n/a	n/a	n/a	n/a	n/a	n/a	n/a	n/a	n/a	n/a	n/a	n/a	n/a	n/a	n/a
	20%	1	1.43	n/a	n/a	n/a	n/a	n/a	n/a	n/a	n/a	n/a	n/a	n/a	n/a	n/a	n/a	n/a	n/a	n/a	n/a
	None	n/a	n/a	n/a	n/a	52	74.29	n/a	n/a	3	4.29	3	4.29	38	54.29	2	2.86	42	60.00	n/a	n/a
	Focal	n/a	n/a	n/a	n/a	17	24.29	n/a	n/a	62	88.57	67	95.71	30	42.86	65	92.86	27	38.57	n/a	n/a
	Moderate	n/a	n/a	n/a	n/a	1	1.43	n/a	n/a	5	7.14	0	0	2	2.86	3	4.29	1	1.43	n/a	n/a
	Severe	n/a	n/a	n/a	n/a	0	0	n/a	n/a	0	0	0	0	0	0	0	0	0	0	n/a	n/a
	1:1	n/a	n/a	n/a	n/a	n/a	n/a	n/a	n/a	n/a	n/a	n/a	n/a	n/a	n/a	n/a	n/a	n/a	n/a	13	40.63
	1:2	n/a	n/a	n/a	n/a	n/a	n/a	n/a	n/a	n/a	n/a	n/a	n/a	n/a	n/a	n/a	n/a	n/a	n/a	13	40.63
	1:3	n/a	n/a	n/a	n/a	n/a	n/a	n/a	n/a	n/a	n/a	n/a	n/a	n/a	n/a	n/a	n/a	n/a	n/a	2	6.25
	1:4	n/a	n/a	n/a	n/a	n/a	n/a	n/a	n/a	n/a	n/a	n/a	n/a	n/a	n/a	n/a	n/a	n/a	n/a	3	9.38
	2:1	n/a	n/a	n/a	n/a	n/a	n/a	n/a	n/a	n/a	n/a	n/a	n/a	n/a	n/a	n/a	n/a	n/a	n/a	1	3.13

IHC: immunohistochemistry; TILs: tumour infiltrating lymphocytes; Freq: frequency; n/a not applicable; TILs: tumour infiltrating lymphocytes; PD-1: Programmed cell death protein 1; PD-L1: Programmed death-ligand 1; PD-L2: Programmed death-ligand 2.

Logistic regression did not identify any statistically significant factors predictive of expression of PD-L1 within tumour cells (Supplementary Table 3). Tumour (T)-stage (Tx vs. T2 stage; Odds Ratio (OR) 0.09 (95%-CI 0.01-0.71); p-value 0.023), nodal (N)-stage (N1 stage vs. N0; OR 9.56 (95%-CI 1.17-77.92); p-value 0.035) and intent of treatment (palliative vs. curative; OR 0.26 (95%-CI 0.08-0.84); p-value 0.024) were identified as potential factors associated with PD-1 expression within TILs in the univariate logistic regression (Supplementary Table 3). Statistical significance was lost in the multivariable analysis (Supplementary Table 4). Neither the administration of systemic treatment (including systemic steroids) before sample acquisition, nor type of sample (primary tumour vs. metastatic site) nor past medical history of systemic inflammatory disease were factors associated with PD-L1 or PD-1 expression.

**Figure 2:**
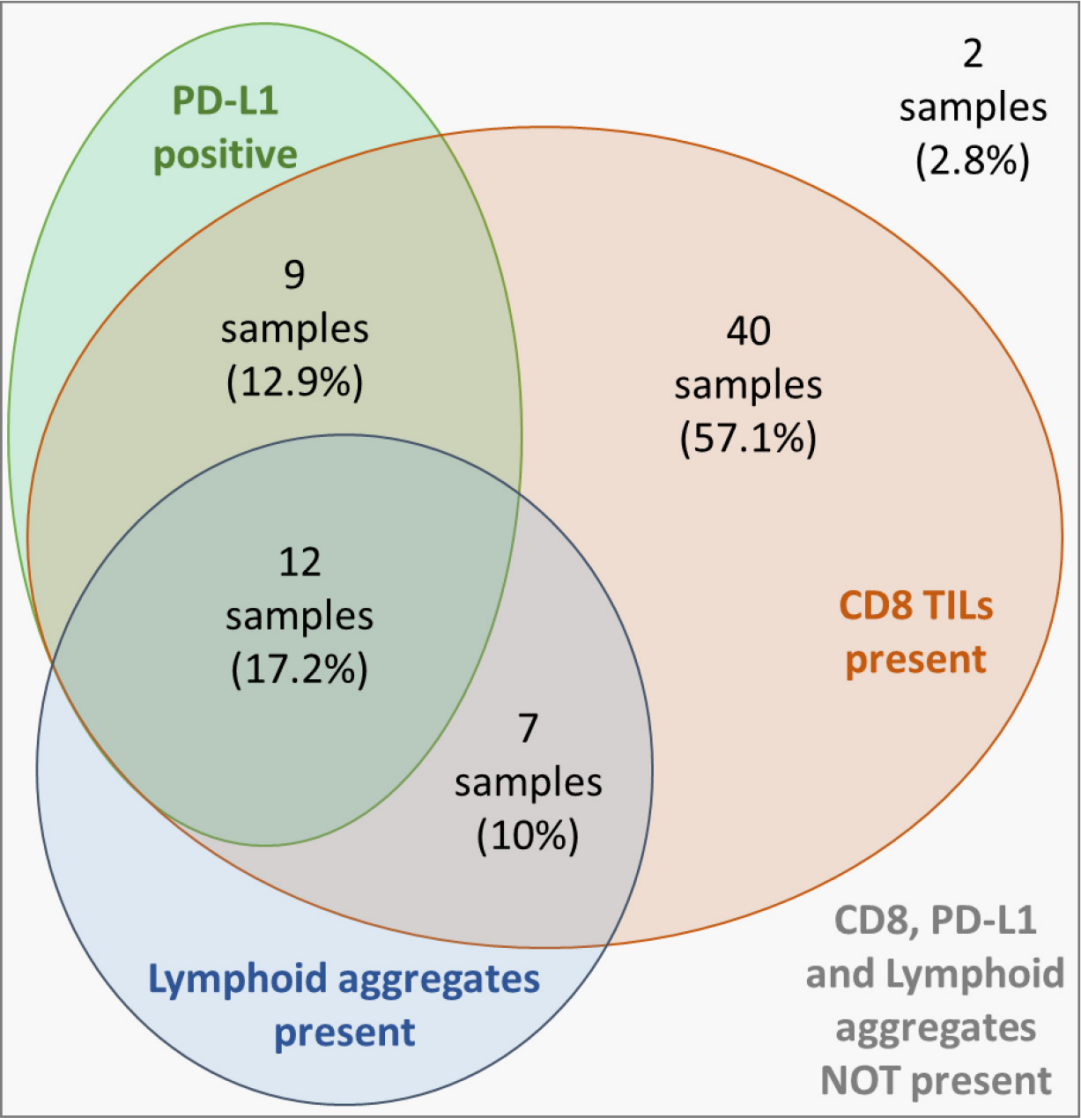
Distribution and overlapping of IHC characteristics PD-L1: Programmed death-ligand 1; IHC: immunohistochemistry.

Co-expression of PD-L1, CD8^+^-TILs and lymphocyte aggregates were assessed within samples. Overall, out of the 70 samples, all three markers were present in 12 samples (17.2%). Forty samples (57.1%) had presence of CD8^+^, in the absence of the other two markers. Twenty-one samples (30%) had expression of PD-L1 together with CD8^+^ infiltration (12 and 9 samples in the presence and absence of lymphocyte aggregates, respectively). See Figure 2 for full detail.

### Impact of IHC results on survival

Expression of PD-L1 within tumour cells did not impact on OS, RFS or PFS (log rank test p-values were 0.4611, 0.4682 and 0.6789, respectively). Similar results were identified for the expression of PD-1 within TILs (log rank test p-values were 0.73414, 0.9642 and 0.8651, respectively).

### Concordance of IHC results and correlation between IHC and RT-qPCR

Out of those patients with repeated samples retrieved (one patient had 4 samples available, and 5 had 2 samples available); good concordance was identified within IHC results (Supplementary Table 5). Out of the total of 126 individual assessments performed within the totality of all samples, 106 (84.1%) were in agreement with results from repeated biopsies.

Samples analysed with IHC were classified as positive for PD-L1 if there was any evidence of expression within tumour cells and/or TILs: 21 samples (30%) showed PD-L1 expression, while 49 samples (70%) did not.

**Figure 3:**
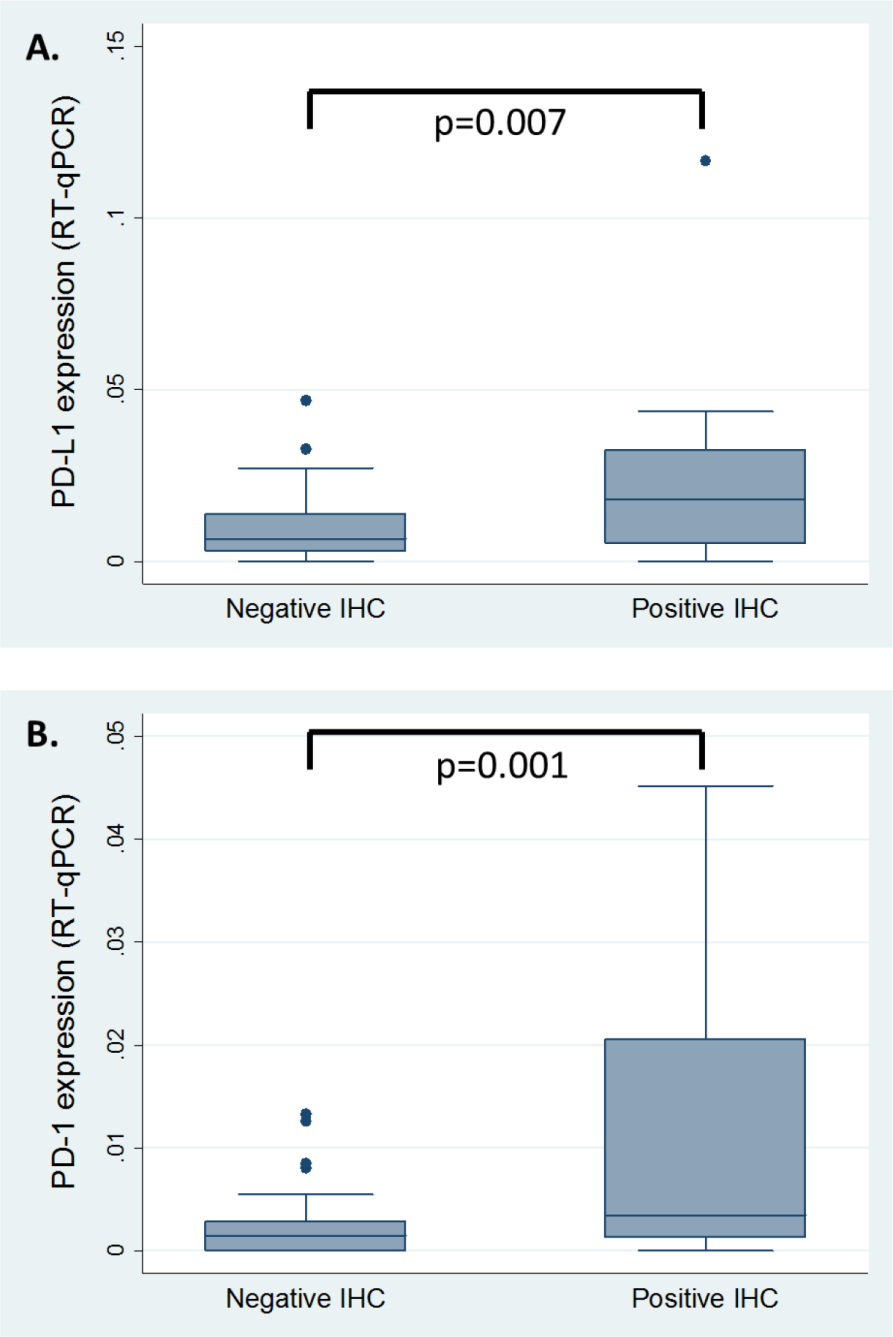
RT-qPCR results correlated with IHC findings Values of expression in RT-qPCR are shown as the difference between the gene of interest and the housekeeping gene. IHC: immunohistochemistry; RT-qPCR: reverse transcription quantitative polymerase chain reaction.

IHC findings were confirmed with RT-qPCR, which showed higher expression levels of PD-L1 (p-value 0.007) and PD-1 (p-value 0.001) in those samples with positive IHC, compared to negative IHC. See Figure 3. However PD-L2 expression was not identified in IHC, median expression of PD-L2 (11.33 x10^-3^ (95%-CI 7.67 x10^-3^ – 14.99 x10^-3^)) by RT-qPCR was similar (t-test p-value 0.2331) to that observed for PD-L1 (13.84 x10^-3^ (95%-CI 9.39 x10^-3^ - 18.28 x10^-3^)), when all samples were analysed.

## DISCUSSION

We have shown that 30% of samples from patients with Wd Si-NETs showed expression of PD-L1 within tumour cells and/or TILs, together with a high rate of TIL presence. Previous series reported a wide range of PD-L1 expression within tumour cells (from 0%[37] to 69%[38]). our findings agree with those of previous studies with respect to the strong presence of lymphocytic infiltrate in Wd Si-NETs (lymphocyte aggregates were present in 27.14% of our samples). In addition, 17.2% of our samples had concomitant presence of CD8^+^, lymphocyte aggregates and PD-L1 expression. Based on our findings and previous evidence supporting the benefit of immunotherapy in tumours rich on immune infiltrate, we believe that immunotherapy compounds warrant further investigation in this patient group [37].

Other series have explored the presence of check-point pathway in neuroendocrine neoplasms (NEN), in both well- and poorly-differentiated tumours from various primary sites (Table 4) [35-38, 39-44]. Within the NEN spectrum, high grade neuroendocrine carcinomas (NECs) such as small cell NEC (including lung primaries)[35] or Merkel cell carcinoma [39] have shown the highest PD-L1 expression. In addition, other groups have shown supremacy of CD8^+^ cells over immune inhibitors (such as T regulatory cells and PD-1 “exhausted” immunocytes) within the global infiltrate in Merkel cell carcinoma samples [45]. Nghiem and colleagues reported high objective response rate (56%; 4 patients had a complete response, and 10 had a partial response) among 25 patients diagnosed with advanced Merkel cell carcinoma treated with pembrolizumab in the first-line setting [20]. Other compounds (such as avelumab) have also had successful results in patients with Merkel cell carcinoma [46]. Within primary lung NENs (well- and poorly-differentiated) high expression of PD-1 and PD-L1 has been reported [40].

**Table 4:** Studies exploring the PD1 pathway in neuroendocrine neoplasms

Tumour differentiation	Subgroup of NETs explored	Reference	Findings
**Poorly-differentiated**	Small cell NEC (any site (n=94); including lung primary (n=61))	Schultheis *et al* [35]	PD-1 expression in stroma 47.9% PD-L1 expression in tumour 0%; PD-L1 expression in stroma 18.5%
	Merkel cell carcinoma (n=21)	Behr *et al* [39]	PD-L1 expression in tumour (8/19); PD-L1 expression in TILs (7/19)
	Merkel cell carcinoma (n=136)	Benhamou *et al* [42]	PD-L1 expression in tumour (57%)
	Large Cell NECs (LCNECs) (n=10)	Fan *et al* [40]	PD-1 expression 80% PD-L1 expression 100%
	Small Cell Lung Cancer (SCLCs) (n=48)	Fan *et al* [40]	PD-1 expression 54.5% PD-L1 expression 50%
	Mixed population of foregut and hindgut primaries (n=15)	Kim *et al* [36]	PD-L1 expression 7/15 (46.7%)
**Mixed populations of Pd and Wd**	Pancreatic NETs (13.7% poorly-differentiated) (n=117)	Saganas *et al* [43]	PD-L1 expression within primary tumours: 58% negative, 42% positive (34% moderately positive, 8% strongly positive) PD-L1 expression within metastatic site: 54% negative, 46% positive (38% moderately positive, 8% strongly positive)
	Mixed population of various gastrointestinal primaries (n=120)	Yinying *et al* *[41]*	PD-1 expression in TILs 55.8% PD-L1 expression in tumour 52.5%
	Lung primaries (11 LCNECs, 49 TC, 5 AC)	Kossai *et al* [44]	PD-L1 expression 0%
**Well-differentiated**	Lung carcinoids (n=22)	Fan *et al* [40]	PD-1 expression 59.1% PD-L1 expression 59.1%
	**Small intestine NETs (n=64)**	Da Silva *et al* [37]	PD-1 expression in stroma 45% PD-L1 expression in tumour 0%; PD-L1 expression in stroma 55% PD-L2 expression in tumour 85% (cytoplasmic location only)
	Pancreatic NETs (n=21)	Da Silva *et al* [37]	PD-1 expression in stroma 47% PD-L1 expression in tumour 11%; PD-L1 expression in stroma 17% PD-L2 expression in tumour 90% (cytoplasmic location only)
	Mixed population of foregut and hindgut primaries (n=17)	Kim et al [36]	PD-L1 expression 0/17 (0%)
	**Midgut NETs (n=32)**	Cives et al [38]	PD-1 expression in TILs observed only in PD-L1 expressing NETs (17/22) PD-L1 expression in tumour (69%)
	**Wd Si-NETs (excluding appendiceal primary) (n=70)**	Lamarca *et al* (current study)	PD-1 expression in TILs 18% PD-L1 expression in tumour 12.83%; PD-L1 expression in TILs 24.29%

Highlighted rows represent those studies/cohorts with similar characteristics to current study population.

NEC: neuroendocrine carcinoma; NETs: neuroendocrine tumours; Pd: poorly-differentiated; Wd: well-differentiated; Si-NETs: small intestine neuroendocrine tumours; TILs: tumour infiltrating lymphocytes; PD-1: Programmed cell death protein 1; PD-L1: Programmed death-ligand 1; n: number of patients; TC: typical carcinoid; AC: atypical carcinoid.

Studies in gastrointestinal NET primaries have shown variable results [36–38]; for example patients with grade 3 tumours showed higher PD-L1 expression (7/15; 46.7%) compared to grade 2 tumours (0/17; 0%) in a series of mixed hindgut and foregut NENs (including well- and poorly-differentiated tumours) [36]. Well-differentiated Si-NETs have been less explored. Only two other series are available, reporting on 32 [38] and 64 [37] patients, respectively. In the series of 32 patients diagnosed with midgut NETs (presented at the ENETS conference in 2016 [38]) expression of PD-L1 was reported in 22 out of 32 human samples (69%; 95%-CI 51-82%). Infiltration of PD-1-positive lymphocytes was observed in 17 of the 22 samples in which PD-L1 expression was identified. In the second series of well-differentiated NETs (64 small intestine primary and 21 pancreatic primary), no membranous expression of PD-L1 was identified among the 64 patients with a small intestine primary (0%) compared to 11% in the 21 patients with pancreatic primaries [37]. This study showed that T-cell tumour infiltrates were frequent in both cohorts; although seemed to be more frequent in pancreatic primary NETs. In addition, a high level of cytoplasmic PD-L2 was observed, of “uncertain significance”. Unfortunately, these studies are only available in abstract form, limiting our ability to assess the methodology and results in detail.

Although other cancer studies (some of them in NETs [41]) have shown a link between PD-L1 expression [47, 48] or presence of TILs [49] with patients’ outcomes, we were not able to confirm such an impact on our series. This is likely to be related to the low number of progressions/deaths (and therefore limited power) for such survival analysis. Thus, longer follow-up is required for confirmation of these results. Moreover, our results did not identify any clinical factors related with higher expression of PD-L1 or PD-1. Of special interest was the fact that we were unable to show higher PD-1/PD-L1 axis activation in samples from patients who had previously received systemic therapy. Within the limitation of a small sample size of pre-treated patients, we found no evidence to suggest that previous treatment increased PD-L1 expression as has been suggested in other scenarios [50].

Immune check-point inhibitors (i.e. anti PD-1 or anti CTLA-4 agents) have revolutionised cancer treatment in some diseases [51, 52]. Unfortunately, it has been challenging to identify *a priori* patients who may benefit from such therapeutic approaches [53, 54]. Initial research was focused on identification of predictive biomarkers such as expression of PD-L1 within tumour cells [47, 55]. It is worth noting that benefit from immunotherapy in the absence of such biomarkers (such as PD-L1) has also been shown and that their presence is not, therefore, an absolute requirement. Data from a randomised clinical trial in patients with advanced melanoma showed that patients benefited from treatment with pembrolizumab, regardless of PD-L1 expression [10]. Similar findings have been reported in other malignancies, such as hepatocellular carcinoma [56]. Other tumour-derived biomarkers have been reported recently, including high tumour mutational load [57], presence of TILs in the tumour microenvironment [58, 59], increased PD-L1 expression on immune cells, high ratio of CD8^+^/CD4^+^ lymphocytes [53] and chromosomal aneuploidy [60]. In addition, research in colorectal cancer has suggested a correlation between the presence of TILs density and the overall number of frame shift mutations [61]. Finally, patient microbiome has also been postulated to impact response to immunotherapy [62].

Based on the above-mentioned research, and the avid interest of the oncology community in developing immunotherapies in a board spectrum of malignancies, multiple research groups have explored such biomarkers in a variety of malignancies, to support clinical trial development in those scenarios [63-65]. Mutational load is known to be “high” in tumours in which immunotherapies have been shown to be effective, such as melanoma, lung and renal cancer [57]. Based on the same rationale, efficacy of check-point inhibitors in solid tumours (colorectal, biliary tract, endometrial, small bowel and gastric) with mismatch-repair deficiencies was explored; a phase II study showed high objective response rate (40%)[66]. Unfortunately, previous studies have described low mutational burden in neuroendocrine tumours [67, 68], which makes this biomarker poorly informative in well-differentiated neuroendocrine malignancies. Mismatch-repair deficiency is also unusual in patients with Wd NETs [69], which are also known for their poor response rate to chemotherapy(8) and good prognosis(1).

In the absence of a reliable discrete biomarker, research has also been focused on a better understanding of the tumoural immune environment as a whole, for identification of tumours more likely to respond to these treatment approaches.

A concept called “tumour immunity continuum” has been proposed [54], in which tumours can be classified in three subgroups according to their inflammation component: “pre-existing immunity”, “excluded infiltrate” and “immunologically ignorant”. The aim of such classification is to identify tumours which may respond favourably to check-point inhibition (“pre-existing immunity”) compared to others which may require a prior conversion to an inflamed phenotype with combined/sequential therapy (“excluded infiltrate” and “immunologically ignorant”).

Other research groups have classified solid tumours based on T-cell infiltration and PD-L1. Teng and colleagues suggested 4 cancer subgroups based on the following criteria [70]: 1) type I (adaptive immune resistance) characterised by presence of both PD-L1 and TIL; 2) type II (immunological ignorance) characterised by the absence of both PD-L1 and TILs; 3) type III (intrinsic induction) which showed expression of PD-L1 in the absence of TILs; and 4) type IV (tolerance/other suppressor pathways) characterised by the presence of TILs, in the absence of PD-L1 expression.

Our results would support defining Wd Si-NETs within the “excluded infiltrate” subtype group as part of the “tumour immunity continuum” classification [54], and also as subtype I (applicable for 30% of samples) or IV (applicable for 70% of samples) within the classification by Teng and colleagues [70]. Thus, immunotherapy, in the form of anti PD-L1 or other alternative approaches (such as metabolites or non-T-cell effector approaches), may be of interest in Wd Si-NETs. It may also be beneficial to combine strategies (combination of immunotherapy agents or combination of immunotherapy with chemotherapy, radiotherapy or targeted agents) for ensuring success of immunotherapy approaches in this scenario. Treatment with chemotherapy and/or radiotherapy can produce tumoural immune suppression [71] and may also lead to the production of neo-antigens [72], and so could be used for “induction” of neo-antigens and immunogenicity with the aim of pursuing immunotherapy approaches afterwards. Thus, immunotherapy development in Wd Si-NETs may also benefit from such “induction” treatment approaches in previously-treated patients (i.e. previously treated with SSA, targeted therapies or chemotherapy). Alternatively, due to the high rate of infiltration of TILs, monotherapy with check-point inhibitors might also be worth exploring.

Up to 2017, few trials were exploring the role of immunotherapy in NENs. Such studies were mainly focused in Merkel cell carcinoma (NCT02584829) due to previous favourable results in this patient population, as discussed above (clinicaltrials.gov; last accessed 12^th^ January 2017). In addition, pembrolizumab efficacy data on the cohort of PD-L1 positive patients diagnosed with NENs recruited into the KEYNOTE-028 study were presented in ESMO 2017 Annual Conference (16 patients) and showed promising results (objective responses were observed in four patients: one patient with pNET and in 3 carcinoid patients)[24]. Over the last 12 months, at least three new studies were opened to recruitment, reflection of the increased interest on developing these agents in NETs (www.clinicaltrials.gov; last accessed 26^th^ July 2017). The humanised anti-PD-1 antibody JS001 is being tested in 40 patients diagnosed with NENs who have progressed to previous treatment (NCT03167853). This study is focused on patients with Ki67 >10%, including both Wd and Pd NENs of any site. Similar patient population is been recruited into a phase II clinical trial with another PD-1 inhibitor (PDR001; NCT02955069). Finally, combination approaches are also been considered; a phase II study is exploring combination of tremelimumab (CTLA-4 inhibitor) and durvalumab (PD-L1 inhibitor) (NCT03095274). Once again, mixed population of NEN patients are being considered for this ongoing study.

The strengths of the current study include the following: all tumour samples were assessed by a pathologist with NET expertise who confirmed eligibility for entry into this study. In addition, in order to maximize the availability of pathological data, we applied strict sample quality criteria for our study excluding 13 out of 75 patients with inadequate samples, and included full-slide samples only rather than tissue micro-arrays. In addition, IHC reading was based on previously accepted international significant scoring systems (i.e. focus on membrane expression for both PD-L1 and PD-L2) securing interpretable results. Confirmation of IHC findings with RT-qPCR also adds to the robustness of our data, in comparison to previously reported series in this patient population [37, 38], which reported IHC data only with the limitations that this implies [73]. Finally, the population’s demographics of patients including in our study were as expected for the population explored, allowing the extrapolation of our results to Wd Si-NETs in daily practice [1], and we excluded patients diagnosed with appendiceal primary tumours, since they are considered to have a different biology to other midgut NETs.

The main limitation of our study is the fact that we were unable to identify any PD-L2 expression, which is most likely to be related to a technical issue with the antibody, even though membranous staining was seen in some of the lymphocytes in the tonsil used as a positive control. Further studies using different clones are needed. The fact that the RT-qPCR identified similar expression to the one shown for the PD-L1 supports this hypothesis. Another limitation was the unavailability of blood biomarkers at the time of biopsy for most of the patients (mainly due to the retrospective design of this study); however the results from nearer the time of the biopsy were retrieved, in some patients the long gap between biopsy and blood test been performed could limit our findings. Limited follow-up (low number of survival events) did also limit our statistical power for survival analysis. Finally, sequencing analysis for assessment of mutation burden and exploring other postulated immunooncology-biomarkers may be worth exploring in this series in the future.

In summary, previous biomarker studies in NENs had suggested that pancreatic NETs and poorly-differentiated NECs were the most promising targets for development of immunotherapy, due to high PD-L1 expression. Our results, together with the fact that previous experience with other forms of immunotherapy (such as IFN) have already shown some benefit in Wd Si-NETs, support the premise that Wd Si-NETs could be a population to target and warrants development of such therapeutic approaches.

## MATERIALS AND METHODS

This was a retrospective study analysing formalin-fixed paraffin embedded (FFPE) archival tissue-samples. This research was approved by the Manchester Cancer Research Centre (MCRC) BioBank Ethics Committee. All patients included in this study provided written informed consent for their tumour samples to be BioBanked for research purposes.

### Patient population and tumour samples

Patients previously-diagnosed with Wd (WHO grades 1-2 [2, 3]) Si-NETs were eligible; all patients had undergone central pathology review (for confirmation of diagnosis and grading according to the WHO classification) performed by a pathologist with an expertise in NETs. Hematoxylin and eosin (H&E) stained slides of all formalin-fixed paraffin-embedded (FFPE) samples from primary tumours and/or metastatic sites were reviewed. Only samples which contained more than 100 evaluable tumour cells and in which tumour content was more than 75% were selected for immunohistochemistry (ICH) and molecular study. Samples with inadequate tumour representation were excluded from the study and such patients were replaced (if possible). Both resection specimens and core-biopsy samples were eligible provided the above-mentioned criteria were fulfilled.

Demographic data, baseline characteristics and treatment details for all patients were collected from institutional records. The Adult Comorbidity Evaluation (ACE)-27 index was employed for assessment of patient comorbidities [74]. Data on multiple blood biomarkers (such as white cell count (WCC), lymphocytes, serum Chromogranin A (CgA) and serum/urine 5-hydroxyindoleacetic acid (5-HIAA)) performed at time of first visit to The Christie NHS Foundation Trust. Survival data was last updated in September 2016. Response to treatment was assessed by Response Evaluation Criteria In Solid Tumours (RECIST) v1.1 [75]; and staging was performed as per European Neuroendocrine Tumour society (ENETS) guidelines for Si-NETs [23].

### Analysis of exploratory biomarkers by IHC in tumour samples

The presence of PD-L1, PD-L2, PD-1 and tumour-infiltrating lymphocytes (TILs) were analysed by immunohistochemistry (IHC). Expression of PD-L1 and PD-L2 were assessed within both tumour cells and TILs, while the expression of PD-1 was explored in TILs only. IHC was performed on full sample slides in order to achieve as much tumour representation per sample as possible, to allow more accurate assessment of both tumour and tumour-infiltrating cells. Haematoxylin and eosin (H&E)-stained slides of all tumours were reviewed. The histologic diagnosis was confirmed, and a representative FFPE tissue block was selected for each tumour. Immunohistochemical studies were performed on deparaffinised 4μm tissue sections using either the Ventana Benchmark Ultra automated staining instrument (Tucson, AZ, USA), CC1 heat-induced epitope retrieval solution or the Leica BondRX (Milton Keynes, UK) and ER2 epitope retrieval buffer according to the manufacturer’s instructions. For phenotyping of TILs, antibodies against CD3, CD4, CD8, CD20 and CD68 were employed (Table 5). Formalin-fixed paraffin-embedded benign tonsil tissue was used as control tissue for all markers, except for PD-L1, for which placental tissue was used as the control tissue. IHC readings were performed by an expert pathologist following previously described [76] scoring systems; such scores employed in this study are summarised in Table 5. Membrane expression only was considered positive for PD-L1 and PD-L2 expression analysis.

**Table 5:** Immunohistochemistry scoring system and primers and probes employed for RT-qPCR

Target	IHC (scoring system)	IHC Antibodies employed	Primers and probes (RT-qPCR)
PD-1	Focal (isolated, <5% of TILs); Moderate (5-50% of TILs); Severe (>50% of TILs)	1:25, clone NAT105; Cell Marque^®^	ccgcacgagggacaatag cagctccccatagtccacag UPL Probe #30
PD-L1	Scored at 5% intervals. Specimens with ≥5% membranous expression were considered “positive”	1:200, clone E1L3N; Cell Signaling Technology^®^	ctactggcatttgctgaacg tgcagccaggtctaattgttt UPL Probe #48
PD-L2	Scored at 5% intervals. Specimens with ≥5% membranous expression were considered “positive”	MAB1224 by R&D Systems^®^	aaagagggaagtgaacagtgct gcttctttagatgtcatatcaggtca UPL Probe #36
CD3, CD4, CD8, CD20, CD68	Semi-quantitative score: None (no immune infiltrates); Focal (mostly perivascular infiltrate with some intratumoural extension); Moderate (prominent extension of immune infiltrates away from perivascular areas and amongst tumour cells); Severe (immune infiltrates obscuring the tumour)	CD3: 1:400; rabbit polyclonal, Dako^®^ CD4: 1:50, clone BC/1F6; MenaPath^®^ CD8: 1:100, clone 4B11; Leica Biosystems^®^ CD20: 1:500, clone L26; Dako^®^ CD68: 1:100, clone PG-M1; Dako^®^	n/a
GAPDH (house-keeping gene)	n/a	n/a	agccacatcgctcagacac gcccaatacgaccaaatcc UPL Probe #60
SDHA (house-keeping gene)	n/a	n/a	cctgtcctatgtggacgttg gttttgtcgatcacgggtct UPL Probe #48

IHC: immunohistochemistry; RT-qPCR: reverse transcription quantitative polymerase chain reaction; n/a applicable; TILs: tumour infiltrating lymphocytes; PD-1: Programmed cell death protein 1; PD-L1: Programmed death-ligand 1; PD-L2: Programmed death-ligand 2.

If patients were found to have more than one sample available, all samples were retrieved in order to assess IHC result agreement between samples.

### Confirmation of IHC results with RT-qPCR

PD-L1, PD-1 and PD-L2 were also assessed with reverse transcription quantitative polymerase chain reaction (RT-qPCR). Total RNA was extracted from 1-3, 5-20 μm thick sections of FFPE tissue using RNeasy FFPE Kit (Qiagen #73504). One μg of total RNA was reverse transcribed using Reverse Transcription High Capacity cDNA Synthesis Kit (Thermo Fisher #437966). Target cDNAs were pre-amplified in 13 PCR cycles using Taqman Preamp Master Mix Kit (Thermo Fisher #4384266). Specific qPCR was carried out on a Quantstudio 5 Real-time PCR instrument (Applied Biosystems) using Taqman Universal Master Mix (Thermo Fisher #4440012) with 10pM/μl gene specific primers and 10 μM probes (Roche Universal Probe Library) (Table 5). Analysis was performed using Quantstudio Design and Analysis software v1.3.1. Two house-keeping genes were initially tested (GAPDH glyceraldehyde-3-phosphate dehydrogenase [GAPDH] and Succinate dehydrogenase complex, subunit A [SDHA]). As SDHA was more consistent across samples than GAPDH, SDHA alone was used as a reference for analysis of findings. Values of expression in RT-qPCR were expressed as the difference between gene of interest and the housekeeping gene.

### Objectives

The aim of this study was to retrospectively explore check-point pathway protein expression (PD-1/PD-L1/PD-L2) and the characteristics of the infiltrating immune cells in Si-NETs with a view of establishing whether there may be potential benefit for targeting this pathway in patients with Wd Si-NETs in future clinical trials. This study also aimed to explore the correlation between pathological findings and clinical and biochemical characteristics. The impact on patient outcomes was also explored.

### Statistical analysis

Stata v.12 software was employed for statistical analysis. Categorical variables were summarised by providing both frequencies and percentages. For continuous variables, median and 95%-confidence interval (95%-CI) was used. Statistical T-test was used for comparison of IHC and RT-qPCR findings. Logistic regression (univariate and multivariable, as applicable) was used for identification of factors predictive for expression of PD-1 or PD-L1 within the TILs or tumour cells, respectively.

Relapse-free survival (RFS) was defined as the time between date of surgery and date of tumour relapse for patients who underwent potentially-curative resection. For patients who received any first-line palliative treatment, progression-free survival (PFS) was measured as the time from starting first-line treatment to the time of disease progression (either radiological or clinical), or the date of death or last follow-up without progression (if the patient was still alive at the end of follow-up). Overall survival (OS) was calculated for all patients as the time from diagnosis to the date of death or last follow-up without death. Median RFS, PFS and OS were estimated by the Kaplan-Meier method. The log-rank test and univariate/multivariable Cox regression models were used for survival analysis as appropriate. Two-sided significance test with a p-value of <0.05 was considered significant for any of the above-mention statistical analyses.

## SUPPLEMENTARY MATERIALS FIGURE AND TABLES




