# Supplementary material for: PD-L1 expression and presence of TILs in small intestinal neuroendocrine tumours
Source: Oncotarget. 2018 Feb 12;9(19):14922–38. doi: 10.18632/oncotarget.24464 (PMC5871087; doi:10.18632/oncotarget.24464)
Supplement: Supplementary file 1 [file oncotarget-09-14922-s001.pdf]

PD-L1 expression and presence of TILs in small intestinal neuroendocrine tumours

SUPPLEMENTARY MATERIALS

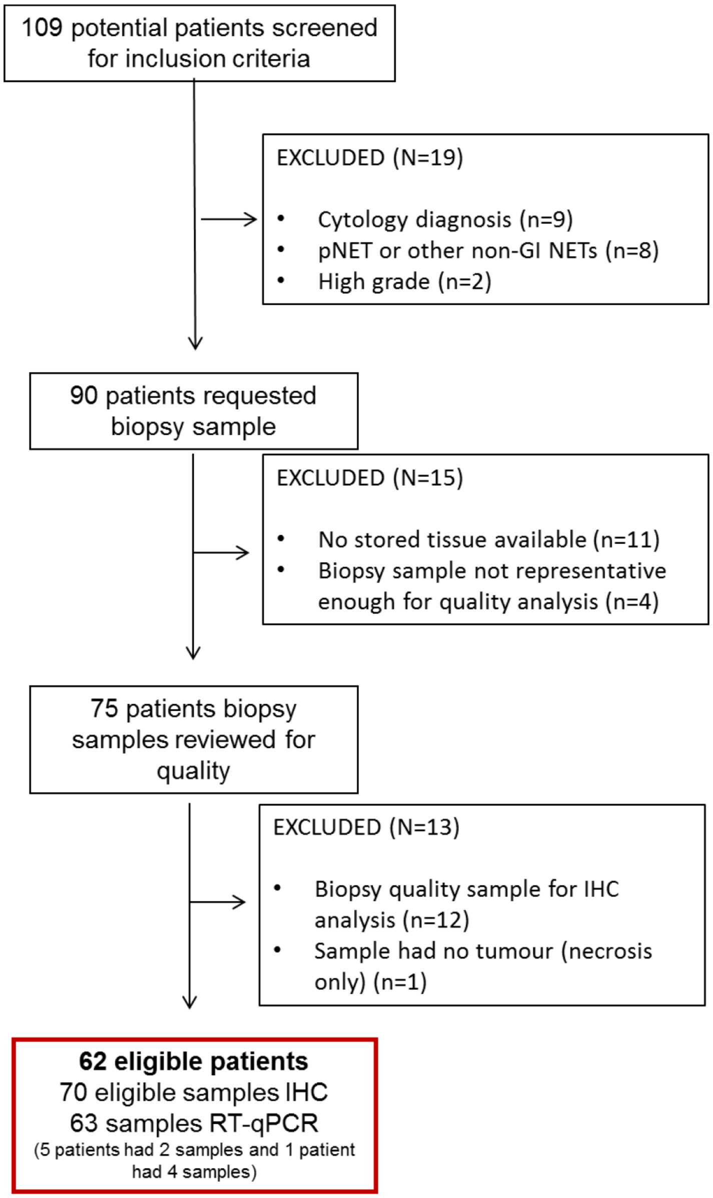

Supplementary Figure 1: Patient flow chart. pNET: pancreatic neuroendocrine tumour; non-GI: non-gastrointestinal; NET: neuroendocrine tumour; IHC: immunohistochemistry; RT-qPCR: reverse transcription quantitative polymerase chain reaction.

Supplementary Table 1: Blood Biomarkers taken at time of first consultation at The Christie NHS Foundation Trust

Variable		Number of observations	
White Cell Count (WCC) ($x10^9/l$)	Median (95%-CI)	70	6.15 (5.8-7.78)
	Median (range)	70	6.15 (3.1-14.2)
Lymphocytes ($x10^9/l$)	Median (95%-CI)	70	1.5 (1.23-1.6)
	Median (range)	70	1.5 (0.3-4.8)
Time between tumour sampling and WCC/ Lymphocyte assessment (months)	Median (95%-CI)	70	1.49 (1.22-1.89)
	Median (range)	70	1.49 (-10.05-119.41)
Chromogranin A (CgA) (ng/mL)	Median (95%-CI)	70	112 (84.91-218.88)
	Median (range)	70	112 (18-45370)
Time between tumour sampling and CgA assessment (months)	Median (95%-CI)	70	2.35 (1.45-5.52)
	Median (range)	70	2.35 (-2.53-76.42)
5-HIAA serum (nmol/L)	Median (95%-CI)	33	124 (90.98-197.72)
	Median (range)	33	124 (30-8510)
Time between tumour sampling and serum 5HIAA assessment (months)	Median (95%-CI)	33	1.48 (1.17-4.02)
	Median (range)	33	1.48 (-2.27-55.40)
5-HIAA urine (umol/24hrs)	Median (95%-CI)	36	29 (21.58-46.84)
	Median (range)	36	29 (12-2171)
Time between tumour sampling and urine 5-HIAA assessment (months)	Median (95%-CI)	36	3.71 (1.76-7.99)
	Median (range)	36	3.71 (-14.65-73.53)

For each variable the median is given with both 95%-CI (upper row) and range (lower row). 5-HIAA: 5-hydroxyindoleacetic acid; 95%-CI: 95% confidence interval.

Supplementary Table 2: Patients' management

**Total of 62 patients**		**Frequency**	**%**
**Resection of primary tumour**	Yes	52	83.87
**Treatment intent at first diagnosis**	Curative resection	32	51.61
	Palliative	30	48.39
**Overall survival (months)**	Median (95%-CI)	195.34 (106.83-not reached)	
**CURATIVE TREATMENT (N=32)**			
**Relapse-free survival (months)**	Median (95%-CI)	62.49 (25.42-142.49)	
**Resection margin resection**	R0	25	78.13
	R1	5	15.63
	Not specified	2	6.24
**Vascular invasion**	Yes	19	59.38
	No	5	15.63
	Not specified	8	25.00
**PALLIATIVE TREATMENT (N=30)**			
**Progression-free survival (months)**	Median (95%-CI)	49.33 (29.23-64.83)	
**First-line treatment**	Chemotherapy	2	6.67
	IFN	3	10.00
	SSA	18	60.00
	Debulking surgery	1	3.33
	Watch and wait	6	20.00
**Best radiological response to first-line treatment**	Stable disease	28	93.33
	Progressive disease	2	6.67
**Second-line treatment**	Yes	19	63.33

Median survivals have been estimated with Kaplan-Meier method. 95%-CI: 95% confidence interval; KM Kaplan-Meier estimation; R0: complete resection; R1: microscopic margins affected; IFN: interferon; SSA: somatostatin analogues.

Supplementary Table 3: Identification of factors predictive of IHC expression of PD-L1 or PD-1 within the tumour cells or TILs, respectively (Univariate logistic regression)

Total of 70 samples		Expression of PD-L1 within tumour cells		Expression of PD-1 within TILs	
		OR (Univariate Logistic Regression)	P-value	OR (Univariate Logistic Regression)	P-value
**Gender**	Male	1 (Ref)	-	1 (Ref)	0
	Female	*Not calculated	n/a	2.33 (0.76-7.16)	0.139
**Age at first diagnosis (years)**	Continuous variable	0.98 (0.93-1.03)	0.394	1.01 (0.96-1.04)	0.966
**Comorbidities (ACE-27)**	None	1 (Ref)	-	1 (Ref)	-
	Mild	1.17 (0.21-6.42)	0.856	1.91 (0.47-7.71)	0.362
	Moderate	2 (0.14-27.99)	0.607	3.33 (0.37-29.39)	0.278
**PMH of systemic inflammatory disease**	No	1 (Ref)	-	1 (Ref)	-
	Yes	2.62 (0.44-15.58)	0.290	3.43 (0.76-15.49)	0.109
**ECOG-PS**	0	1 (Ref)	-	1 (Ref)	-
	1	4.04 (0.77-21.17)	0.099	1.30 (4.38-3.89)	0.633
	2	*Not calculated	n/a	*Not calculated	n/a
	3	*Not calculated	n/a	*Not calculated	n/a
**Carcinoid syndrome**	Yes (Any symptom)	0.34 (0.06-1.75)	0.196	1.09 (0.37-3.21)	0.875
	Flushing (Yes)	*Not calculated	n/a	2.12 (0.67-6.67)	0.199
	Diarrhoea (Yes)	0.54 (0.10-2.86)	0.472	0.49 (0.14-1.73)	0.271
	Wheezing	*Not calculated	n/a	0.55 (0.06-5.08)	0.600
**TNM (ENETS)**	II	1 (Ref)	-	1 (Ref)	-
	III	0.27 (0.02-4.01)	0.343	2.83 (0.93-8.60)	0.066
	IV	0.27 (0.02-3.55)	0.319	*Not calculated	n/a
**T (primary tumour)**	2	1 (Ref)	-	1 (Ref)	-
	3	1.88 (0.18-19.53)	0.599	0.38 (0.07-2.16)	0.272
	4	Not calculated	n/a	0.21 (0.03-1.25)	0.087
	X	1.13 (0.09-13.03)	0.925	0.09 (0.01-0.71)	**0.023**
**N (lymph node)**	0	1 (Ref)	-	1 (Ref)	-
	1	0.74 (0.17-3.33)	0.699	9.56 (1.17-77.92)	**0.035**
	X	*Not calculated	n/a	*Not calculated	n/a
**M (distant metastases)**	0	1 (Ref)	-	1 (Ref)	-
	1	0.81 (0.19-3.33)	0.771	0.42 (0.14-1.26)	0.123
**Number of sites of metastases**	1	1 (Ref)	-	1 (Ref)	-
	2	*Not calculated	n/a	0.25 (0.03-2.28)	0.217
	3	1.83 (0.15-22.37)	0.635	*Not calculated	n/a
**Treatment intent at first diagnosis**	Curative resection	1 (Ref)	-	1 (Ref)	-
	Palliative	0.73 (0.18-2.96)	0.654	0.26 (0.08-0.84)	**0.024**
**Vascular invasion**	Yes	1 (Ref)	-	1 (Ref)	-
	No	0.24 (0.5-1.18)	0.080	0.74 (0.18-3.01)	0.675
	Not specified	0.10 (0.01-1.04)	0.054	0.47 (0.09-2.38)	0.362
**White Cell Count (WCC)**	Continuous variable	0.99 (0.74-1.32)	0.939	1.01 (0.80-1.25)	0.977
**Lymphocytes**	Continuous variable	0.77 (0.26-2.23)	0.624	1.06 (0.52-2.17)	0.864
**Chromogranin A (CgA)**	Continuous variable	0.99 (0.98-1.01)	0.751	0.99 (0.98-1.01)	0.778
**5HIAA serum**	Continuous variable	0.99 (0.98-1.01)	0.577	0.99 (0.98-1.01)	0.859
**5HIAA urine**	Continuous variable	0.95 (0.85-1.05)	0.322	1.001 (0.99-1.01)	0.325
**Grade**	Grade 1	1 (Ref)	-	1 (Ref)	-
	Grade 2	0.22 (0.03-1.89)	0.168	0.73 (0.22-2.36)	0.595
**Ki67**	Continuous variable	0.89 (0.58-1.37)	0.593	0.43 (0.14-1.26)	0.123
**Systemic treatment before sample acquisition**	No	1 (Ref)	-	1 (Ref)	-
	Yes	1.05 (0.19-5.69)	0.959	1.62 (0.47-5.58)	0.449
**Sample type**	Primary tumour	1 (Ref)	-	1 (Ref)	-
	Metastatic site	0.54 (0.10-2.86)	0.472	0.49 (0.14-1.73)	0.271

*Not calculated: refers to subgroups which could not be explored due to colineality.

IHC: immunohistochemistry; Ref: reference; OR: odds ratio; n/a: not applicable; ECOG-PS: Eastern Cooperative Oncology Group Performance Status score; 95%-CI: 95% confidence interval; NET: neuroendocrine tumour; PMH: past medical history; ACE-27: Adult Comorbidity Evaluation (ACE)-27 index; ENETS: European Neuroendocrine Tumour Society; X: not evaluated; 5-HIAA: 5-hydroxyindoleacetic acid; TILs: tumour infiltrating lymphocytes; PD-1: Programmed cell death protein 1; PD-L1: Programmed death-ligand 1.

Supplementary Table 4: Multivariable logistic regression for IHC prediction of PD-1 expression within TILs; those variables statistically significant within the univariate analysis (Supplementary Material 2) were included

**Total of 70 samples**		**Expression of PD-1 within TILs (Univariate analysis)**			**Expression of PD-1 within TILs (Multivariable analysis; 68 patients)**	
		**Number of observations**	**OR (Univariate Logistic Regression)**	**P-value**	**OR (Multivariable Logistic Regression)**	**P-value**
**T (primary tumour)**	2	70	1 (Ref)	-	1 (Ref)	-
	3		0.38 (0.07-2.16)	0.272	0.45 (0.07-2.84)	0.398
	4		0.21 (0.03-1.25)	0.087	0.31 (0.05-1.98)	0.215
	X		0.09 (0.01-0.71)	**0.023**	0.56 (0.04-7.23)	**0.656**
**N (lymph node)**	0	68	1 (Ref)	-	1 (Ref)	-
	1		9.56 (1.17-77.92)	**0.035**	8.44 (0.69-102.70)	**0.094**
	X		*Not calculated	n/a	*Not calculated	n/a
**Approach at first diagnosis**	Curative resection	70	1 (Ref)	-	1 (Ref)	-
	Palliative		0.26 (0.08-0.84)	**0.024**	0.33 (0.87-1.28)	**0.109**

*Not calculated: refers to subgroups which could not be explored due to colineality.
IHC: immunohistochemistry; 95%-CI: 95% confidence interval; X: not evaluated; TILS: tumour infiltrating lymphocytes; OR: odds ratio; n/a: not applicable; TILs: tumour infiltrating lymphocytes; PD-1: Programmed cell death protein 1; PD-L1: Programmed death-ligand 1.

Supplementary Table 5: IHC findings within patients with multiple samples available

Out of all the comparative assessments performed, 84.1% were in agreement (green fields represent full concordance between samples)

See Supplementary File 1
