# Supplementary material for: PD-L1 expression and presence of TILs in small intestinal neuroendocrine tumours
Source: Oncotarget. 2018 Feb 12;9(19):14922–38. doi: 10.18632/oncotarget.24464 (PMC5871087; doi:10.18632/oncotarget.24464)
Supplement: Supplementary file 2 [file oncotarget-09-14922-s002.docx]

## Supplementary Table 5: IHC findings within patients with multiple samples available. Out of all the comparative assessments performed, 84.1% were in agreement (green fields represent full concordance between samples).

| Patient | Sample | Sample description | Grade (Ki-67) | PD-L1 expression (tumour cells) | PD-L1 expression (TILs) | PD-L1 expression (Any) | PD-1 expression | CD3 | CD68 | CD4 | CD8 | CD20 | CD4/CD8 ratio (applicable only for samples with CD4 and CD8 expression) |
| --- | --- | --- | --- | --- | --- | --- | --- | --- | --- | --- | --- | --- | --- |
| **Patient A** | Sample 1 | Liver metastasectomy, 1998, previous 5FU chemotherapy | Grade 2 (Ki67 not available) | No | No | No | No | Yes; Focal | Yes; Focal | No | Yes; Focal | No | n/a |
|  | Sample 2 | Liver metastasectomy, 1998, previous 5FU chemotherapy | Grade 2 (Ki67 not available) | No | No | No | No | Yes; Focal | Yes; Focal | No | Yes; Focal | No | n/a |
|  | Sample 3 | Liver metastasectomy, 1998, previous 5FU chemotherapy | Grade 2 (Ki67 not available) | No | No | No | No | Yes; Focal | Yes; Focal | No | Yes; Focal | No | n/a |
|  | Sample 4 | Liver metastasectomy, 1998, previous 5FU chemotherapy | Grade 2 (Ki67 not available) | No | No | No | No | Yes; Focal | Yes; Focal | No | Yes; Focal | No | n/a |
| **Patient B** | Sample 1 | Liver metastasectomy; 2013; no previous treatment | Grade 1 (Ki67 2%) | No | Yes; 5% | Yes | No | Yes; Focal | Yes; Focal | Yes; Focal | Yes; Focal | Yes; Focal | 1:1 |
|  | Sample 2 | Liver metastasectomy; 2014, previous treatment with Lanreotide | Grade 1 (Ki67 2%) | No | Yes; 5% | Yes | No | Yes; Focal | Yes; Focal | Yes; Focal | Yes; Focal | Yes; Focal | 1:1 |
| **Patient C** | Sample 1 | Primary tumour 2014; no previous treatment | Grade 1 (Ki67 1%) | Yes; 5% | Yes; 5% | Yes | Yes; Focal | Yes; Focal | Yes; Focal | Yes; Focal | Yes; Focal | Yes; Focal | 1:2 |
|  | Sample 2 | Liver metastasectomy 2015; previous treatment with Octreotide | Grade 1 (Ki67 1%) | No | No | No | Yes; Focal | Yes; Focal | Yes; Focal | Yes; Focal | Yes; Focal | Yes; Focal | 2:1 |
| **Patient D** | Sample 1 | Primary tumour 2008; no previous systemic treatment | Grade 1 (Ki67 2%) | No | No | No | Yes; focal | Yes; Focal | Yes; Focal | Yes; Focal | Yes; Focal | Yes; Focal | 1:1 |
|  | Sample 2 | Peritoneal metastasis 2012; no previous systemic treatment | Grade 2 (Ki67 5%) | No | No | No | No | Yes; Focal | Yes; Focal | No | Yes; Focal | No | n/a |
| **Patient E** | Sample 1 | Primary tumour 2007; no previous systemic treatment | Grade 1 (Ki67 2%) | Yes; 5% | No | Yes | No | Yes; Focal | Yes; Focal | Yes; Focal | Yes; Focal | No | 1:1 |
|  | Sample 2 | Liver metastasectomy 2008; no previous systemic treatment | Grade 1 (Ki67 2%) | Yes; 20% | No | Yes | No | Yes; Focal | Yes; Focal | No | Yes; Focal | No | n/a |
| **Patient F** | Sample 1 | Primary tumour 2006; no previous systemic treatment | Grade 1 (Ki67 1%) | No | No | No | No | Yes; Focal | Yes; Focal | No | Yes; Focal | Yes; Focal | n/a |
|  | Sample 2 | Peritoneal metastasis 2012; previous treatment with Interferon | Grade 1 (Ki67 2%) | No | No | No | No | Yes; Focal | Yes; Focal | Yes; Focal | Yes; Focal | No | 1:1 |

TILs: tumour infiltrating lymphocytes; PD-1: Programmed cell death protein 1; PD-L1: Programmed death-ligand 1; 5-FU: 5-fluorouracile.
